# Contributions of GeneXpert^®^ to TB diagnosis in Myanmar

**DOI:** 10.5588/ijtld.22.0009

**Published:** 2022-09-01

**Authors:** Z. Burger, H. T. Aung, M. Seifert, T. T. Mar, V. Harris, R. E. Colman, T. C. Rodwell, S. T. Aung

**Affiliations:** 1 University of California San Diego, La Jolla, CA, USA; 2 Clinton Health Access Initiative, Yangon, Myanmar; 3 Ministry of Health and Sports, Naypyitaw, Myanmar; 4 Foundation for Innovative New Diagnostics, Geneva, Switzerland

**Keywords:** tuberculosis, Xpert, Myanmar

## Abstract

**BACKGROUND:**

Xpert^®^ MTB/RIF, a rapid, molecular TB diagnostic assay, can detect *Mycobacterium tuberculosis* and rifampin resistance directly from clinical sputum samples in <2 h with high sensitivity and specificity. The added diagnostic value of Xpert over smear microscopy at a national level in Myanmar has not been previously reported.

**METHODS:**

We evaluated 339,358 Xpert and demographic records captured from January 2015 to December 2018 as part of the Myanmar National TB Program Data Utilization and Connectivity Project to examine the additional diagnostic yield of Xpert relative to smear for the detection of *M. tuberculosis* for TB diagnosis in Myanmar, with a focus on people living with HIV (PLHIV) and sample type.

**RESULTS:**

Use of Xpert increased TB case detection by 40% compared to smear microscopy results. Among PLHIV, use of Xpert increased TB case detection by almost 100% compared to smear microscopy results.

**CONCLUSION:**

Xpert testing identified more patients with TB than smear microscopy alone, particularly in cohorts with significant proportions of PLHIV. The use of Xpert as a screening tool in countries with a high burden of TB could lead to significantly increased diagnosis of TB at a regional and national level.

## Abstract (fr)

**CONTEXTE:**

Xpert® MTB/RIF, un test de diagnostic moléculaire rapide de la TB, permet de détecter *Mycobacterium tuberculosis* et la résistance à la rifampicine directement à partir d’échantillons d’expectorations cliniques en <2 h avec une sensibilité et une spécificité élevées. La valeur diagnostique ajoutée d’Xpert par rapport à la microscopie à frottis au niveau national au Myanmar n’a pas été rapportée auparavant.

**MÉTHODES:**

Nous avons évalué 339 358 enregistrements Xpert et démographiques saisis de janvier 2015 à décembre 2018 dans le cadre du projet d’utilisation et de connectivité des données du programme national de lutte contre la TB du Myanmar afin d’examiner le rendement diagnostique supplémentaire d’Xpert par rapport au frottis pour la détection de *M. tuberculosis* pour le diagnostic de la TB au Myanmar, en mettant l’accent sur les personnes vivant avec le VIH (PLVIH) et le type d’échantillon.

**RÉSULTATS:**

L’utilisation d’Xpert a augmenté de 40% la détection des cas de TB par rapport aux résultats de la microscopie à frottis. Chez les PLVIH, l’utilisation d’Xpert a augmenté la détection des cas de TB de presque 100% par rapport aux résultats de l’examen microscopique des frottis.

**CONCLUSION:**

Le test Xpert a permis d’identifier plus de patients atteints de TB que la microscopie à frottis seule, en particulier dans les cohortes comportant une proportion importante de PLVIH. L’utilisation d’Xpert comme outil de dépistage dans les pays à forte charge de TB pourrait conduire à une augmentation significative du diagnostic de la TB au niveau régional et national.

Myanmar (population: over 54 million) ranks among the 30 highest TB burden countries as classified by the WHO and has one of the highest rates of TB-HIV coinfection in the world.^1^ Myanmar has made significant progress in reducing new TB infections in recent years through a rigorous program of testing and treatment developed by the Myanmar National TB Programme (NTP), and recently met the WHO milestone of a 20% reduction in TB incidence as part of the End TB Strategy set in 2015.^1^ The program is centered around a national network of Xpert^®^ MTB/RIF instruments (Cepheid, Sunnyvale, CA, USA) used for the diagnosis of TB and detection of rifampin (RIF) resistant TB.

Xpert is a rapid nucleic-acid amplification test (NAAT) that has dramatically improved the detection and diagnosis of *Mycobacterium tuberculosis* globally. Xpert is recommended for both diagnosis of TB and identification of RIF resistance by the WHO,^2^ but quantification of the diagnostic impact of implementing Xpert at a national level in Myanmar has not been previously reported.

Despite the improved performance of Xpert diagnosis over smear microscopy, smear microscopy remains the foundation of TB diagnosis in many low- and middle-income countries due to its simplicity and low cost. However, smear microscopy has relatively low sensitivity (20–60%),^3^,^4^ especially in people living with HIV (PLHIV 9–39%),^5^–^7^ who have a lower bacillary load in their sputum samples compared with HIV-negative individuals.^8^,^9^ Due to Xpert’s high sensitivity, its use among PLHIV has consistently increased the detection of TB in these populations.^10^–^12^

Few large-scale studies of high-burden countries have assessed the added value of Xpert for bacterial confirmation of TB at a national scale.^13^,^14^ Our study objective was primarily to compare the diagnostic yield of Xpert with traditional smear microscopy for the detection of *M. tuberculosis* for TB diagnosis in Myanmar, with a focus on PLHIV. Additionally, given that Xpert can be used to test a variety of clinical sample types, our secondary objective was to examine Xpert results for the detection of *M. tuberculosis* in blood, cerebrospinal fluid (CSF), gastric lavage, and saliva.

## METHODS

### Study data

In our analysis, we used Xpert and demographic data captured from January 2015 to December 2018 as part of the Myanmar Data Utilization and Connectivity Project. This project was developed by the Myanmar NTP with the assistance of FIND (Foundation for Innovative New Diagnostics, Geneva, Switzerland), and has been described previously in Seifert et al.^15^ Briefly, Xpert results from clinical sites distributed across the country were automatically generated by the instrument and collected centrally using the GxAlert connectivity solution from System-One (Northampton, MA, USA); associated demographic data were entered into an electronic data collection system at each clinical site. While the decision-making process for determining which individuals received Xpert testing evolved over the project period as part of the continued rollout of Xpert testing in Myanmar, the final inclusion criteria for Xpert testing were individuals who were at risk for TB (i.e., presumptive TB patients) and 1) individuals at risk of having drug-resistant TB; 2) individuals living with HIV; 3) individuals with diabetes; 4) persons with a history of TB treatment; and 5) children under 15 who were able to produce sputum. Individuals with positive TB smears at 2 or 5 months after treatment initiation were also referred for additional Xpert testing to detect potential RIF resistance during treatment. All samples were evaluated with direct smear microscopy using either fluorescence or Ziehl-Neelsen staining techniques, depending on site testing capacity.

Data were stripped of all unique patient identifiers prior to this analysis. The final dataset included Xpert test records from 87 clinical sites across Myanmar and included the following variables: test date, Xpert assay result (including both *M. tuberculosis* and RIF resistance detection), smear status, sex, age category, HIV status, and for a subset of patients, clinical sample type.

### Analyses and statistics

The data presented here are based on standard-of-care data from the Myanmar NTP. The WHO considers Xpert to be a “reference” level test, with a pooled sensitivity and specificity of 85% and 98%, respectively (sensitivity among smear- and culture-positive samples was 98%; this was 67% in smear-negative, culture-positive samples).^16^,^17^ Therefore, results that were positive on Xpert in this study were considered to be “true-positives” for our analyses.

We excluded any Xpert sample that did not have an accompanying positive or negative smear result. In our sub-analyses of Xpert and smear results by HIV status (Table 1), any sample that did not have an HIV status or age was excluded from that analysis. In Table 1, adults were defined as age ≥18 years, while the pediatric population was defined as age <18 years. All analyses were performed using STATA/SE v16.1 (Stata Corp, College Station, TX, USA).

**Table 1:** Added value of Xpert based on HIV status

	HIV-positive			HIV-negative		
	
	Total^*^ *n* (%)	Adult *n* (%)	Pediatric *n* (%)	Total^*^ *n* (%)	Adult *n* (%)	Pediatric *n* (%)
Xpert MTB-positive	13,063	12,909	112	71,534	69,497	1,695
2015	2,559	2,539	17	6,007	5,882	111
2016	2,954	2,911	32	11,030	10,727	270
2017	3,540	3,497	32	23,398	22,639	597
2018	4,010	3,962	31	31,099	30,249	717
Xpert MTB-positive, smear-positive	6,560 (50.2)	6,479 (50.2)	58 (51.8)	55,165 (77.1)	53,660 (77.2)	1,235 (72.9)
2015	1,202 (47.0)	1,187 (46.8)	13 (76.5)	3,713 (61.8)	3,642 (61.9)	61 (55.0)
2016	1,537 (52.0)	1,519 (52.2)	12 (37.5)	7,913 (71.7)	7,710 (71.9)	183 (67.8)
2017	1,757 (49.6)	1,733 (49.6)	17 (53.1)	18,126 (77.5)	17,563 (77.6)	430 (72.0)
2018	2,064 (51.5)	2,040 (51.5)	16 (51.6)	25,413 (81.7)	24,745 (81.8)	561(78.2)

* Includes records without age recorded.

### Ethics

This study received ethics approval from the Ethics Review Committee, Department of Medical Research, Ministry of Health and Sports, Naypyitaw, Republic of the Union of Myanmar (Approval number: Ethics/DMR/2018/155) for analysis of study data and was classified as exempt from review by the University of California San Diego Institutional Review Board, La Jolla, CA, USA.

## RESULTS

A total of 339,358 Xpert results were collected from 2015 through 2018 by the Myanmar Data Utilization and Connectivity Project. Records without smear results were removed from the dataset (*n* = 9,095). *M. tuberculosis* was detected in 141,021 samples (44.8%) using Xpert, with 158,699 (50.4%) *M. tuberculosis-*negative results and 15,093 (4.8%) results recorded as “Error,” “Invalid,” or “No Result.” Approximately 17% of the study population was HIV-positive, 41% HIV-negative, and 42% had unknown HIV status. Among the HIV-positive individuals, 23.9% were Xpert *M. tuberculosis-*positive, while 55.5% of the HIV-negative individuals and 43% of the HIV-unknown individuals were Xpert *M. tuberculosis-*positive. See Table 2.

**Table 2:** Characteristics of the study population stratified by HIV status

	HIV status		

	Positive (*n* = 54,732) *n* (%)	Negative (*n* = 128,931) *n* (%)	Unknown (*n* = 131,150) *n* (%)
Xpert result			
MTB-positive	13,063 (23.9)	71,534 (55.5)	56,424 (43.0)
Rifampicin-resistant	1,113 (8.5)	6,363 (8.9)	6,336 (11.2)
*M. tuberculosis* invalid/error/no result	3,024 (5.5)	5,730 (4.4)	6,339 (4.8)
Smear result			
Smear-positive	7,077 (12.9)	59,431 (46.1)	42,001 (32.0)
Smear-negative	47,655 (87.1)	69,500 (53.9)	89,149 (68.0)
Age, years, median [IQR] (*n* = 1,638 missing)	36 [30–44] (*n* = 147 missing)	45 [32–59] (*n* = 611 missing)	47 [32–60] (*n* = 880 missing)
Sex			
Female	17,900 (32.7)	42,447 (32.9)	46,759 (35.7)
Male	36,627 (66.9)	85,891 (66.6)	83,619 (63.8)
Unknown/missing	205 (0.4)	593 (0.5)	772 (0.6)
Test year (% by year)			
2015	9,739 (23.9)	11,552 (28.3)	19,473 (47.8)
2016	11,739 (19.4)	21,022 (34.7)	27,886 (46.0)
2017	15,507 (16.4)	40,143 (42.5)	38,817 (41.1)
2018	17,747 (14.9)	56,214 (47.3)	44,974 (37.8)

IQR = interquartile range.

The mean age of the individuals studied was 44.1 years (standard deviation 17.6, interquartile range 31–57). There was a statistically significant age difference between individuals recorded as HIV-positive, HIV-negative or HIV-unknown as determined using one-way analysis of variance (*P* < 0.0001). A Tukey post-hoc test revealed that HIV-positive individuals were significantly younger than individuals recorded as HIV-negative or -unknown (*P* < 0.0001).

The number of individuals tested using Xpert over time tripled from 40,764 in 2015 to 118,935 in 2018, as additional Xpert testing sites across Myanmar were continuously added to the project. Overall, the absolute number of HIV-positive individuals tested between 2015 and 2018 increased year over year. However, the proportion of HIV-positive individuals tested decreased from 2015 through 2018 (Table 2). The RIF prevalence reported in Table 2 reflects the RIF resistance detected within the study population rather than the prevalence of RIF resistance among all TB cases in Myanmar.

A total of 141,021 samples were Xpert *M. tuberculosis-*positive. Of these positive samples, 100,834 (71.5%) were smear-positive and 40,187 (28.5%) were smear-negative. Therefore, smear captured only 71.5% (100,834/141,021) of likely TB cases using Xpert as reference. Additionally, 4,277 (1.3%) samples were classified as smear-positive and *M. tuberculosis-*negative using Xpert. Xpert-negative, smear-positive patients underwent further evaluation, and those with abnormal radiographic findings were clinically diagnosed with TB and treated according to the NTP protocol. The added value of Xpert is even more pronounced among PLHIV. Smear captured only 50.2% (6,469/12,909) of the total Xpert-positive cases among adults living with HIV (Table 1). Similarly, among HIV-positive children, smear captured only 51.8% (58/112) of TB cases detected using Xpert. Overall, using Xpert allowed healthcare workers to detect almost twice as many adults living with HIV and co-infected with *M. tuberculosis* than smear (12,909 vs. 6,479), and almost twice as many children living with HIV and TB than smear alone (112 vs. 58).

Xpert detection of *M. tuberculosis* was also higher than smear detection of *M. tuberculosis* among the HIV-negative populations. Smear captured only 77.2% of TB cases among HIV-negative adults and 72.9% of TB cases among HIV-negative children (Table 1). Overall, Xpert increased TB case detection by 40% compared to smear alone.

There were 21,730 samples labelled by sample type in our database and the vast majority (92.6%) were sputum samples. Other sample types collected included blood, gastric lavage, CSF, and saliva (Table 3). Xpert was used effectively to detect TB across a broad range of sample types, with only a very small percentage of samples documented to have indeterminate results (error, invalid, or no result) across all sample type categories. The added value of Xpert was highest among CSF and gastric lavage samples. Gastric lavage sampling was performed exclusively among children with an average age of 4 years. Four effusion samples and one pus sample were excluded from the analysis due to low sample size.

**Table 3:** Details of Xpert-positives among diverse sample types

Sample type/total of sample type tested	Blood (*n* = 24) *n* (%)	CSF (*n* = 204) *n* (%)	Gastric lavage (*n* = 437) *n* (%)	Saliva (*n* = 911) *n* (%)
Xpert MTB-positive	13 (54.2)	13 (6.4)	19 (4.4)	237 (26.0)
Xpert indeterminate (error, invalid or no result)	0 (0.0)	5 (2.5)	4 (0.9)	30 (3.3)
Xpert MTB-positive, smear-positive, *n* (smear-positive/Xpert MTB-positive)	12 (92.3)	0 (0.0)	2 (10.5)	97 (40.9)

CSF = cerebrospinal fluid.

## DISCUSSION

The overall goal of our study was to compare the diagnostic yield of Xpert to traditional smear microscopy for detection of *M. tuberculosis* for TB diagnosis in Myanmar. Over 28% of the almost 141,000 TB cases detected in Myanmar from 2015 through 2018 were smear-negative, but Xpert-positive. With Myanmar’s steady incorporation of network-linked Xpert instruments across a growing number of clinical sites, 40% more cases of TB were diagnosed compared to what would have been diagnosed with traditional smear microscopy alone. The introduction of Xpert had an even more profound effect among PLHIV; almost 100% more TB cases among the total adult and pediatric HIV-positive populations were diagnosed using Xpert compared to traditional smear microscopy. These are cases that would have otherwise been missed if smear alone was used for the detection of TB in Myanmar (Table 1).

Our results support previous research that has shown that sputum smear microscopy is negative in more than half of patients with HIV-associated TB.^8^,^10^,^18^ This is critical at a programmatic level, as undetected and untreated TB disease is more likely to advance to disseminated disease in PLHIV.^19^ Xpert’s superior diagnostic capability across a variety of different sample types has significant potential to decrease morbidity and mortality among these patients through earlier TB detection and treatment.^4^,^17^,^20^

Of the 21,730 samples labelled by sample type in our database, the vast majority (92.6%) were sputum samples. Other sample types collected included blood, gastric lavage, CSF, and saliva (Table 3). The test indeterminate rate of Xpert across all sample types was low (<4%), suggesting that Xpert can be reliably used to detect *M. tuberculosis* across a diverse range of sample types when sputum is not available. The added value of Xpert in non-sputum sample types was highly variable, ranging from 8% in blood to 100% in CSF. These findings from the Myanmar NTP suggest that Xpert can be effectively used to great affect among pediatric populations (who often require gastric lavage as a clinical sample^21^), and PLHIV who can have higher rates of extrapulmonary TB disease requiring non-pulmonary samples for Xpert diagnosis. While we had no data on the reasons for different sample types being collected for each patient, we assumed based on NTP protocols, that alternative samples were collected only when sputum samples were not obtainable or when extrapulmonary disease was indicated. Regardless of the reason for alternative sample collection, different sample sizes made it difficult to interpret the added value of Xpert by sample type.

There were several limitations to this study. The data were collected for programmatic purposes and were not collected under research conditions. The most important limitation this introduced was the lack of a common TB reference culture standard against which we could have estimated the comparative sensitivity and specificity of both Xpert and microscopy. To estimate the potential diagnostic advantage of Xpert vs. microscopy we had to assume Xpert-positive cases were “true-positives”. While there is a possibility that a small fraction of these Xpert-positive results are “false-positives,” it is unlikely to be significant, as Xpert is a WHO-endorsed TB reference standard and it has been well documented that Xpert is highly sensitive and specific for TB.^17^

Despite the limitations of this study, these data from the Myanmar NTP, which include almost 150,000 TB cases over 4 years, indicate that Xpert can have a substantial diagnostic advantage over acid-fast bacilli smear microscopy alone. With Xpert, the Myanmar NTP was able to capture 40% more TB cases in the general population and almost 100% more TB cases among PLHIV than if they had continued to rely on smear alone. There are, however, valid pragmatic arguments for the continued strengthening of microscopy networks globally due to concerns about the cost, sustainability and reach of molecular techniques at all levels of the healthcare system.^22^ We support this view that existing smear microscopy networks should not be abandoned but rather enhanced by the adoption of national testing network using linked molecular diagnostic tests, as countries that continue to use smear microscopy exclusively for the diagnosis of TB risk missing a substantial portion of their total TB cases. Incorporating Xpert testing in high TB burden countries, particularly if their populations have significant proportions of PLHIV, could lead to improved TB detection, earlier treatment initiation and lower mortality for patients.^23^–^27^
